# Differences in the Head Roll Test, Bow and Lean Test, and Null Plane between Persistent and Transient Geotropic Direction-Changing Positional Nystagmus

**DOI:** 10.3390/jcm9010073

**Published:** 2019-12-27

**Authors:** Sung Kyun Kim, Sung Won Li, Seok Min Hong

**Affiliations:** Department of Otorhinolaryngology-Head and Neck Surgery, Dongtan Sacred Heart Hospital, College of Medicine, Hallym University, #7 Keunjaebong-gil, Gyeonggi-do, Hwaseong 18450, Korea; newearera@hallym.or.kr (S.K.K.); sw9731@hallym.or.kr (S.W.L.)

**Keywords:** direction-changing positional nystagmus, head roll test, null plane, mechanism

## Abstract

Background: Persistent geotropic direction-changing positional nystagmus (DCPN) has the characteristics of cupulopathy, but its underlying pathogenesis is not known. We investigated the relationship of the results of the head roll test, bow and lean test, and side of the null plane between persistent and transient geotropic DCPN to determine the lesion side of persistent geotropic DCPN and understand its mechanism. Methods: We enrolled 25 patients with persistent geotropic DCPN and 41 with transient geotropic DCPN. We compared the results of the head roll test, bow and lean test, and side of the null plane between the two groups. Results: The rates of bowing and leaning nystagmus were significantly higher in the persistent DCPN group. Only 16.0% of the persistent DCPN patients had stronger nystagmus in the head roll test and the null plane on the same side. The rates of the direction of bowing nystagmus in the bow and lean test and stronger nystagmus in the head roll test on the same side were also significantly lower in persistent DCPN than in transient DCPN. Conclusion: It was difficult to determine the lesion side in persistent geotropic DCPN using the direction of stronger nystagmus in the head roll test and null plane when the direction of the stronger nystagmus and null plane were opposite. Further study is needed to understand the position of the cupula according to head rotation and the anatomical position in persistent geotropic DCPN.

## 1. Introduction

Persistent geotropic direction-changing positional nystagmus (DCPN) that differs from typical transient geotropic DCPN (canalolithiasis) has been proposed as a variant of horizontal semicircular canal-benign paroxysmal positional vertigo (HSCC-BPPV). In persistent geotropic DCPN, the specific gravity of the cupula is lower than that of the surrounding endolymph, which either activates or inhibits hair cells under the cupula according to the head position in the gravitational plane [1,2,3,4]. Possible mechanisms for persistent geotropic DCPN have been suggested, but its underlying pathogenesis is not known [4,5,6].

Transient persistent DCPN does not show a null plane because it is caused by free floating otolith. On the other hand, the presence of the null plane and duration of geotropic DCPN in the head roll test are important findings for diagnosing persistent geotropic DCPN and determining its laterality [7,8]. Theories hold that the side of stronger nystagmus in the head roll test and side of the null plane must be the same, as has been reported in several studies [4,8,9]. In some cases of persistent geotropic DCPN, however, the stronger side in the head roll test and side of the null plane were opposite, which makes it difficult to determine the side of the affected semicircular canal, affecting treatment.

The bow and lean test improves the accuracy of lateralization in HSCC-BPPV [10]. Bowing and leaning nystagmus is also seen in patients with persistent geotropic DCPN, and the direction of the bowing nystagmus is concordant with the side of the null plane [11].

Therefore, this study investigated the results of the head roll test, bow and lean test, and side of the null plane between persistent and transient geotropic DCPN to help determine the lesion side in persistent geotropic DCPN and understand its mechanism.

## 2. Materials and Methods

### 2.1. Subjects and Clinical Variables

Our study was approved by our Institutional Review Board (IRB no. 2016-06-199). The subjects were 25 patients with persistent geotropic DCPN (8 males, 17 females; mean age 54.1 years) and 41 patients with transient geotropic DCPN (10 males, 31 females; mean age 49.6 years) who were diagnosed at our clinic from August 2018 to July 2019. We excluded the patients with vestibular neuritis or sudden sensorineural hearing loss and performed neurologic examination in all patients and magnetic resonance imaging (MRI) in case of suspected central cause.

### 2.2. Persistent and Transient DCPN

All patients had a detailed medical history taken and audiological and vestibular evaluations performed in the same sequence, including video-nystagmography and caloric tests. After examining spontaneous nystagmus, when geotropic DCPN was observed in the head roll test, we tried to determine the null plane. A null plane at which the nystagmus disappears was identified when the patient’s head was turned slightly to either side in the supine position. Then we performed the bow and lean test with the patients in a seated position. The head was bent forward 90° for a least 1 min and then tilted backwards 60° for at least 1 min. Eye movement was examined using goggles fitted to an infrared camera (Otometrics, ICS Chartr 200, Taastrup, Denmark).

The diagnostic criteria for persistent geotropic DCPN were the presence of geotropic DCPN for more than 2 min in the head roll test and the presence of a null plane, while the criterion for transient geotropic DCPN was the presence of geotropic DCPN for less than 1 min in the head roll test.

We compared the incidence of bowing and leaning nystagmus and analyzed the relationship among the stronger side of nystagmus in the head roll test, the directions of bowing and leaning nystagmus, and the side of the null plane in patients with persistent and transient geotropic DCPN.

### 2.3. Statistical Analyses

The clinical parameters of the groups were compared using independent *t*-tests, chi-square tests, or Fisher’s exact test. The chi-square test was used to compare the incidence of bowing and leaning nystagmus and the direction of bowing nystagmus in the bow and lean test and the direction of stronger nystagmus in the head roll test between persistent and transient geotropic DCPN. All statistical tests were conducted using SPSS for Windows (ver. 21.0; SPSS, Chicago, IL, USA) and *p*-values <0.05 were deemed to indicate statistical significance.

## 3. Results

### 3.1. Demographic Data

Table 1 summarizes the demographic and clinical data of the groups with persistent and transient geotropic DCPN. The two groups were matched in terms of age, sex, and lesion side, and the parameters did not differ significantly between the two groups.

### 3.2. Bow and Lean Test and Null Plane

Bowing and leaning nystagmus in the bow and lean test were observed in 21 of 25 patients (84.0%) with persistent geotropic DCPN. The bowing and leaning nystagmus were on the opposite sides in all patients. In comparison, in transient geotropic DCPN, 19 of 41 patients (46.3%) had bowing or leaning nystagmus. The difference between the two groups was significant (*p* = 0.004). Seventeen patients had both bowing and leaning nystagmus, which was on the opposite sides in all 17, and two patients had only leaning nystagmus (Table 2).

### 3.3. Analyses of Nystagmus Direction in the Bow and Lean Test, Head Roll Test, and Null Plane

In persistent DCPN patients with null plane, the directions of stronger nystagmus in the head roll test and the null plane were the same in only 4 of 25 (16.0%) patients and the direction of bowing nystagmus in the bow and lean test and the side of null plane were the same in 20 of 21 (95.2%) patients. In the analyses of the direction of bowing nystagmus in the bow and lean test and the direction of stronger nystagmus in the head roll test, the two forms of nystagmus were in the same direction in only 7 of 21 (33.3%) patients with bowing nystagmus in persistent geotropic DCPN and 14 of 19 (73.7%) patients with transient geotropic DCPN (*p* = 0.014) (Table 3).

## 4. Discussion

Previous studies have reported that the clinical data and resolution time differed between persistent and transient geotropic DCPN and suggested the mechanisms differed [6,8,12]. In one study, the conventional repositioning maneuver used to treat geotropic DCPN (HSCC-BPPV) was ineffective for patients with persistent geotropic DCPN [13]. According to Ewald’s second law, the lesion side in DCPN is the side showing more intense nystagmus during the head roll test in geotropic DCPN (canalolithiasis type) and less intense in apogeotropic DCPN (cupulolithiasis type) [14]. In apogeotropic DCPN, a null plane may help to distinguish the lesion side [15]. Because persistent geotropic DCPN is a cupulopathy, a null plane exists, as in apogeotropic DCPN, and the lesion side has been in the direction of stronger nystagmus in the head roll test and null plane in many studies [6,8,13,16]. However, we found that the direction of stronger nystagmus in the head roll test and the side of null plane matched in only 16.0% of cases.

**Figure 1:**
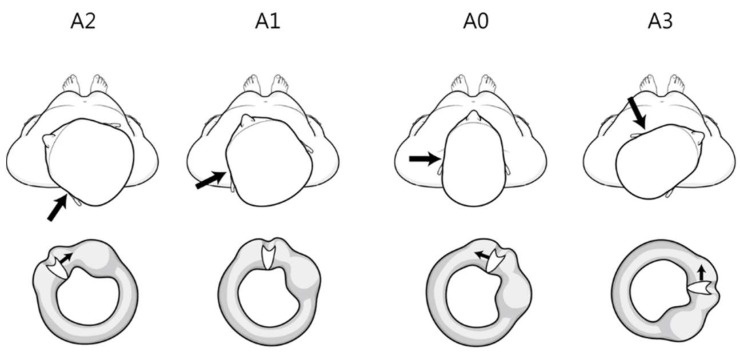
Theoretical findings of the position of the cupula according to the head rotation angle during the head roll test in left persistent direction-changing positional nystagmus. The large and small arrows indicate the lesion side and direction of cupula movement, respectively. The left horizontal canal and cupula viewed from the front. A0 (supine position) cupula medial to lateral position. A1 (null plane) rotated 20–30° to the left (lesion side). Cupula parallel to the vertical axis. A2 (head turning to the left, lesion side) rotated an additional 40–50°. Cupula 40–50° downward from the vertical axis. A3 (head turning to the right, opposite side of the lesion) Cupula 90–100° to the vertical axis.

First, the position of the cupula according to the head rotation angle in the head roll test can explain the mismatch. In persistent DCPN, the position of the cupula with head rotation in the head roll test differs from that in transient DCPN. When the head is rotated from the supine position 20–30° to the lesion side, the cupula is parallel to the direction of gravity (null plane) and the nystagmus disappears. If the roll test is done to the lesion side, an additional 40–50° of rotation is done (many patients can rotate their heads about 70° to the left and right in the head roll test). Eventually, the cupula is about 40–50° downward with respect to the vertical axis. However, when the roll test is performed on the side opposite the lesion, the cupula is about 90–100° relative to the vertical axis (Figure 1). 

In patients with DCPN with a null plane, at 90° rotation from the null plane, both vertigo and nystagmus are the most intense [16]. Therefore, contrary to our expectations, the nystagmus could be stronger when turning to the opposite side in the head roll test in patients with persistent DCPN. That is, the lesion side is the direction of the null plane, not the direction of stronger nystagmus in the head roll test.

**Figure 2:**
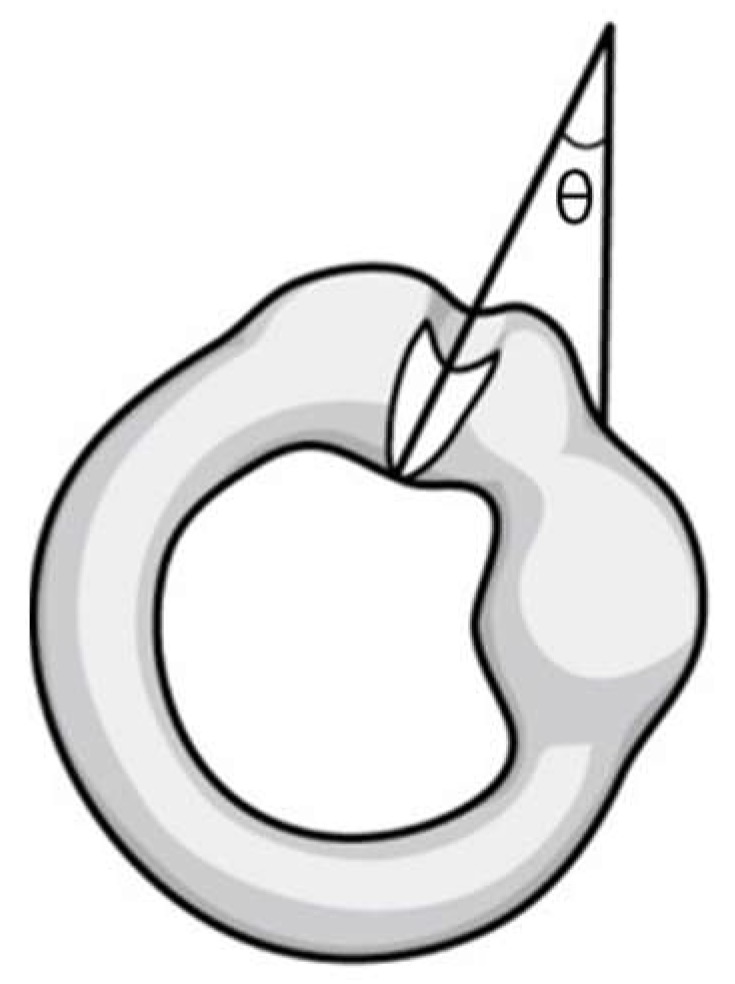
Left horizontal canal and cupula in the supine position (viewed from the front). θ: Angle between the cupula and perpendicular line.

Second, we considered the anatomical direction of the cupula. There is little information on the anatomy of the cupula in HSCC. Curthoys et al. reported the dimensions of HSCC in the human temporal bone in a post-mortem study [17]. In the paper, the direction of the cupula in HSCC is depicted medial to lateral. That is, the cupula in HSCC tilts laterally from the sagittal plane and the angle (θ) is formed between the cupula and perpendicular line (Figure 2). 

**Figure 3:**
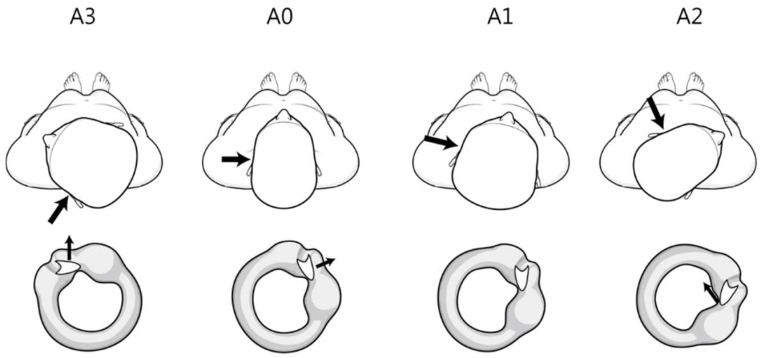
Theoretical findings of the anatomical direction of the cupula (from lateral to medial) during the head roll test in left persistent direction-changing positional nystagmus. The large and small arrows indicate the lesion side and direction of cupula movement, respectively. The left horizontal canal and cupula viewed from the front. A0 (supine position) cupula lateral to medial position. A1 (null plane) rotated 20–30° to the right (opposite side of the lesion). Cupula parallel to the vertical axis A2 (head turning to the right, opposite side of the lesion) rotated an additional 40–50°. Cupula 40–50° downward from the vertical axis. A3 (head turning to the left, lesion side) Cupula 90–100° to the vertical axis.

Many studies of persistent geotropic DCPN have cited the anatomy of the cupula from Curthoys et al. and described the direction of the cupula as ‘medial to lateral’; however, Curthoys et al. did not mention the direction. Bergenius et al. suggested that when judging the affected side, knowledge of the angle of the plane of the cupula relative to the sagittal plane is crucial and that the plane of the cupula in HSCC is approximately parallel to the anterior SCC, but is has not been checked or confirmed [2]. If so, the side of the null plane is opposite the lesion and opposite the side of stronger nystagmus in the head roll test (Figure 3). That is, the lesion side is the direction of stronger nystagmus in the head roll test.

We performed the bow and lean test to help identify the lesion side in persistent geotropic DCPN. In this group, the higher expression rate of the bow and lean test than in the transient group was thought to be due to the characteristics of cupulopathy, with less fatigue caused by the bow and lean test after the head roll test and identification of the null plane.

If the direction of the cupula is medial to lateral, the direction of bowing nystagmus should be the same as the direction of stronger nystagmus in the head roll test because it causes ampullopetal flow of endolymph and nystagmus on the lesion side while the cupula moves in the direction opposite gravity while bowing. However, the concordance of the two tests was only 33.3%. This discrepancy can be explained by the two reasons mentioned above. First, during bowing, the cupula is in an ampullopetal direction, and the direction of bowing nystagmus is the lesion side. As already explained, due to the position of the cupula in the head roll test, the direction of the stronger nystagmus is opposite to the lesion. Second, the discrepancy between these two tests can be explained by considering the direction of the cupula as lateral to medial. During bowing, the cupula is in an ampullofugal direction, which causes nystagmus on the side opposite the lesion. This direction is opposite that of the head roll test and in the same direction as the null plane.

The above two explanations are possible when the head roll test can clearly determine the lesion side according to Ewald’s second law. When the direction of stronger nystagmus is unclear in the head roll test, the direction of stronger nystagmus could be the same as the direction of the null plane or bowing nystagmus.

Though we did not include it in the results, there were four patients of geotropic DCPN associated with inner ear disease (vestibular neuritis or idiopathic sudden sensorineural hearing loss with canal paresis). All four were persistent DCPN. In all four, the direction of the null plane was opposite the stronger nystagmus in the head roll test. Because geotropic DCPN occurred with inner ear disease, the lesion side of persistent geotropic DCPN was likely to be the same as the side of the inner ear disease. If so, neither the null plane nor stronger nystagmus in the head roll test was completely concordant with the lesion side. This suggests that the determination of lesion side in persistent geotropic DCPN cannot be explained by single theory. However, we excluded them in analysis because they had a vestibular imbalance, which might affect nystagmus that occurred during the bow and lean test or head roll test.

## 5. Conclusions

It was difficult to determine the side of persistent geotropic DCPN from the directions of the null plane or stronger nystagmus in the head roll test when the directions of the null plane and head roll test were opposite. This could be explained by the position of the cupula according to the head rotation angle in the head roll test or the anatomical direction of the cupula in persistent geotropic DCPN.

## Figures and Tables

**Table 1:** Demographic findings between persistent and transient geotropic DCPN (direction-changing positional nystagmus).

	Persistent DCPN, *n* = 25	Transient DCPN, *n* = 41
Age, y, mean age SD (range)	54.1 ± 13.4 (38−77)	49.6 ± 14.7(10−76)
Sex ratio: men/women (*n*)	8:17	10:31
lesion side (*n*) *	7:17 †	13:28

* side of stronger nystagmus in head roll test. † non-localized lesion side in one patient.

**Table 2:** Result of bow and lean test (BLT) and null plane between persistent and transient geotropic DCPN (direction-changing positional nystagmus).

	Persistent DCPN	Transient DCPN
BLT (%) *	21/25 (84.0)	19/41 (46.3%)
BN (+), LN (+)	21	17
Opposite side	21	17
Same side	0	0
BN(−), LN(+)	0	2

BN = bowing nystagmus; LN = leaning nystagmus, * *p* < 0.05.

**Table 3:** Analysis of bow and lean test (BLT), head roll test (HRT) and the side of null plane between persistent and transient geotropic DCPN (direction-changing positional nystagmus).

	Persistent DCPN, *n* = 25	Transient DCPN, *n* = 41
HRT: Null plane (%)	4/25 (16.0)	-
BLT: Null plane (%)	20/21 (95.2)	-
BLT: HRT (%) *	7/21 (33.3)	14/19 (73.7)

HRT: Null plane = case in which the direction of strong nystagmus in head roll test and the side of null plane during head roll in supine position are the same, BLT: Null plane = case in which the direction of bowing nystagmus in bow and lean test and the side of null plane during head roll in supine position are the same, BLT: HRT = case in which the direction of bowing nystagmus in bow and lean test and the direction of stronger nystagmus in head roll test are the same. * *p* < 0.05.
